# Some Aspects of Intestinal Obstruction

**Published:** 1908-06-27

**Authors:** 


### Surgery.

### SOME ASPECTS OF INTESTINAL OBSTRUCTION.

### CARCINOMA OF THE INTESTINE.

Our knowledge of the ætiology of carcinoma of the intestine is in the same state as our knowledge of the ætiology of carcinoma in general. To put it, perhaps, a little bluntly we really know nothing about it if we exclude the more or less established fact that it tends to appear at certain points of election which correspond to those portions of the alimentary canal which are narrower than the rest, so that the mucous membrane is subjected to a constant irritation. And this factor of irritation is also supposed to account for the frequency with which healed intestinal ulcers are found to be the seat of a carcinoma.

Sex is of little importance in determining its incidence; the majority of statistics tend to show that it is slightly more common in males than in females, but only so slightly that no importance can be attached to it.

Like carcinoma elsewhere it usually makes its appearance between the ages of forty and sixty-five. But every year an increasing number of cases of intestinal carcinoma in young patients is now reported. This may be due to the fact that the abdomen is now more frequently opened for exploratory purposes, and the tumours so found are more systematically examined with the microscope. The writer has had under his care a boy aged fourteen with a spheroidal celled carcinoma of the descending colon.

Carcinoma may occur anywhere in the intestinal canal from the pylorus to the anus. It is nearly always primary. Metastatic carcinoma in the intestine is uncommon and, when it does occur, rarely gives rise to symptoms of obstruction. On the other hand primary carcinoma of the intestine is very prone to give rise to metastatic deposits. The organs affected are, in their order of relative frequency, mesenteric lymphatic glands, liver, peritoneum, and omentum. Other viscera are less commonly attacked. Statistics show that its frequency decreases from below upwards—*i.e.* it is most common in the rectum; next in order comes the large intestine, while, with the exception of the pylorus, carcinoma of the small bowel is comparatively rare.

It is admitted by histologists that primary carcinoma of the intestine always starts as a columnar celled growth from the epithelium lining the crypts of Lieberkühn, except in the duodenum, where it begins in the epithelium of Brunner's glands. Its microscopical characteristics resemble those of glandular carcinoma elsewhere. The epithelial cells of Lieberkühn's glands proliferate and are arranged in layers: the lumen of the gland is obliterated, and the gland itself now consists of a number of cylinders of epithelium. Sooner or later the epithelial cells make their way through the limiting membrane which confines them, and from that moment the growth becomes a carcinoma. The cells spread, and run riot in the submucous layer, reproducing their original structure as they do so, with the hideous rapidity which is characteristic of malignant disease.

While the essential histology of the condition at its origin is in all cases identical, the macroscopical appearances are subject to great variation. When the growth is seen in its early stages, it is found to appear as a very small nodule occupying a circumscribed portion of the intestinal wall. The nodule may develop into a single massive tumour as large as an adult fist, or larger, or it may protrude into the lumen of the bowel, like a polypoid or cauliflower mass; or it may extend over a large surface of the wall, so that the affected portion becomes converted into a rigid tube. Finally it may extend round the bowel and form an annular stricture. The last form is very often found where the carcinoma has appeared in the site of a healed ulcer. In this variety the stroma is generally in excess of the cellular element, and the growth is consequently hard. The mucous membrane over it soon gives way, and the irritation to which it is now exposed will cause it to ulcerate, slough, or bleed.

Any of these forms of carcinoma may cause intestinal obstruction, but it is the annular variety which does so most constantly. The appearance of the gut at the site of the stricture is very typical. It appears to be suddenly constricted as if a piece of string had been drawn tightly round it. The peritoneum in the neighbourhood is thickened, and the gut itself is often fixed by adhesions. The degree of stenosis seen from within is variable. The narrowed part may admit the thumb or be so constricted as barely to admit a goose-quill. The surface of the growth is nearly always ulcerated, and occasionally the patency of the stenosed bowel may be re-established by the ulceration and sloughing of the surface of the growth; but if the patient survive it is almost certain to close up again.

If the affected portion of bowel be removed post-mortem and examined from above and below, the appearance presented is most characteristic. The gut narrows abruptly and the stenosed part looks like the waist of an hour glass. As Treves describes it, "the inner surface of the ring is ragged and sloughy looking. Above and below the stricture the growth spreads out upon the bowel. It forms an ulcerated surface, raised, spongy-looking, and often comparatively smooth, which terminates in a pronounced edge that stands up above the normal mucous membrane as a raised, everted, rounded, and hardish margin."

The clinical picture presented by carcinoma of the intestine resembles in the main that produced by any other organic stenosis; but in carcinoma the syndrome may, and will, be modified by the site and the structure of the growth and by the implication of other organs, to such an extent that one can recognise symptoms peculiar to carcinoma as it affects the various portions of the intestinal tract. Some of the methods at our disposal for diagnosing the site of the lesion in intestinal obstruction—whether that lesion be carcinomatous or otherwise—have already been described in this journal (August 17, 1907).

